# Epigenetic modifications in abdominal aortic aneurysms: from basic to clinical

**DOI:** 10.3389/fcvm.2024.1394889

**Published:** 2024-06-04

**Authors:** YuChen Liu, XiaoYun Sun, Zhen Gou, ZhenKun Deng, YunRui Zhang, PingPing Zhao, Wei Sun, Yang Bai, YuChen Jing

**Affiliations:** ^1^ Department of Clinical Pharmacology, School of Pharmacy, China Medical University, Shenyang, Liaoning, China; ^2^ Department of Vascular Surgery, The First Affiliated Hospital of China Medical University, Shenyang, China

**Keywords:** abdominal aortic aneurysm, epigenetics, DNA methylation, histone modification, non-coding RNA

## Abstract

Abdominal Aortic Aneurysm (AAA) is a disease characterized by localized dilation of the abdominal aorta, involving multiple factors in its occurrence and development, ultimately leading to vessel rupture and severe bleeding. AAA has a high mortality rate, and there is a lack of targeted therapeutic drugs. Epigenetic regulation plays a crucial role in AAA, and the treatment of AAA in the epigenetic field may involve a series of related genes and pathways. Abnormal expression of these genes may be a key factor in the occurrence of the disease and could potentially serve as promising therapeutic targets. Understanding the epigenetic regulation of AAA is of significant importance in revealing the mechanisms underlying the disease and identifying new therapeutic targets. This knowledge can contribute to offering AAA patients better clinical treatment options beyond surgery. This review systematically explores various aspects of epigenetic regulation in AAA, including DNA methylation, histone modification, non-coding RNA, and RNA modification. The analysis of the roles of these regulatory mechanisms, along with the identification of relevant genes and pathways associated with AAA, is discussed comprehensively. Additionally, a comprehensive discussion is provided on existing treatment strategies and prospects for epigenetics-based treatments, offering insights for future clinical interventions.

## 1 Introduction

Abdominal Aortic Aneurysm (AAA) are aneurysm-like bulbous protrusions that develop on the wall of the abdominal aorta, patients with AAA typically do not exhibit significant symptoms in the early stages of the disease. There may be mild pulsation or pressure sensations, but these symptoms are not pronounced. It is only when the aneurysm reaches a certain ruptured size that the rupture of the abdominal aorta becomes the patient's sole apparent symptom, leading to life-threatening abdominal hemorrhage (1, 2). Annually, at least 150,000 people die from AAA, with the majority of deaths attributed to aneurysm rupture, carrying a high mortality rate of approximately 70%–80% after rupture (3, 4).

Under normal circumstances, the aorta of a healthy individual is elastic. However, due to the combined effects of genetic, environmental, and various complex factors, the vascular wall in the abdominal aortic region may weaken. In such cases, the gradual force of blood against the damaged vascular wall initiates the formation of AAA. As the disease progresses, the arterial wall becomes weak and swollen, unable to withstand the stress of blood flow within the artery, ultimately leading to the rupture of the AAA (2, 5). Clinical treatment for AAA generally involves surgical methods, with Open Aneurysm Repair (OAR) or Endovascular Aneurysm Repair (EVAR) being commonly used. OAR is suitable for patients with a longer life expectancy and lower morbidity rates. Although EVAR has, to some extent, reduced short-term mortality after AAA repair, operated patients experience an increased burden on the aortic vasculature, leading to an elevated risk of death (6). However, surgery has its drawbacks, as it requires complex screening and risk assessment before AAA surgery. Postoperatively, there may be instances of endoleaks, and the long-term care entails significant economic costs (7). Therefore, we may need to find a more reliable or universal treatment than surgery for AAA. An alternative treatment method is pharmaceutical intervention. Risk factors for AAA in diagnostic screening include hypertension, atherosclerosis, diabetes, etc, medications targeting these risk factors related diseases may help slow the progression of AAA, reducing the risk of rupture. In light of these information, the results of preclinical studies in animal models suggest that statins, antihypertensive drugs, doxycycline, metformin, and others have delaying effect on AAA progression (8–10). However, the three main AAA models currently available are elastase, CaCl2, and angiotensin II (AngII)/apolipoprotein E (AapoE)-deficient mouse models, each of which has defects that may not translate the findings to human AAA, and there is currently insufficient evidence from multiple clinical drug trials to conclusively prove the complete efficacy of these drugs in treatment (11, 12). At the same time, these drugs may have side effects in the treatment of AAA, and may have contraindications, for example, statins can cause related muscle symptoms (13), and β-blockers in antihypertensive drugs may reduce cardiac output and vasodilation (14), which indicate that the search for a new treatment option is essential.

Over the past few decades, with the accumulation of molecular biology knowledge and the rapid development of molecular biology techniques, epigenetics, as a significant branch of genetics, has gradually gained widespread attention and in-depth research. Its role in physiological and pathological conditions has sparked a keen interest in personalized medicine (15). Research in the cardiovascular field indicates that the three major epigenetic modifications—DNA methylation, histone modification, and non-coding RNA modification—may play a crucial role in the occurrence and development of cardiovascular diseases (16). Recent studies suggest a correlation between epigenetics and the pathogenesis of cardiovascular diseases and AAA (17–19), including the role of DNA methyltransferases, the modification of histones, the mechanisms of non-coding RNA and RNA modification, which will be described in detail below. The progress of AAA is a complex and multi-layered issue, involving inflammation, matrix metalloproteinase (MMP) activation, oxidative stress, intraluminal thrombosis, smooth muscle apoptosis, and extracellular matrix (ECM) changes, among others, the characteristics of AAA described above may be closely related to epigenetic regulatory outcomes (1, 20). By elucidating the current research on epigenetics related to AAA and discussing the potential involvement of epigenetic regulation in AAA progression, rupture, and repair, this paper aims to provide insights and perspectives for identifying new therapeutic targets.

## 2 Epigenetic mechanism of abdominal aortic aneurysm

**Figure 1:**
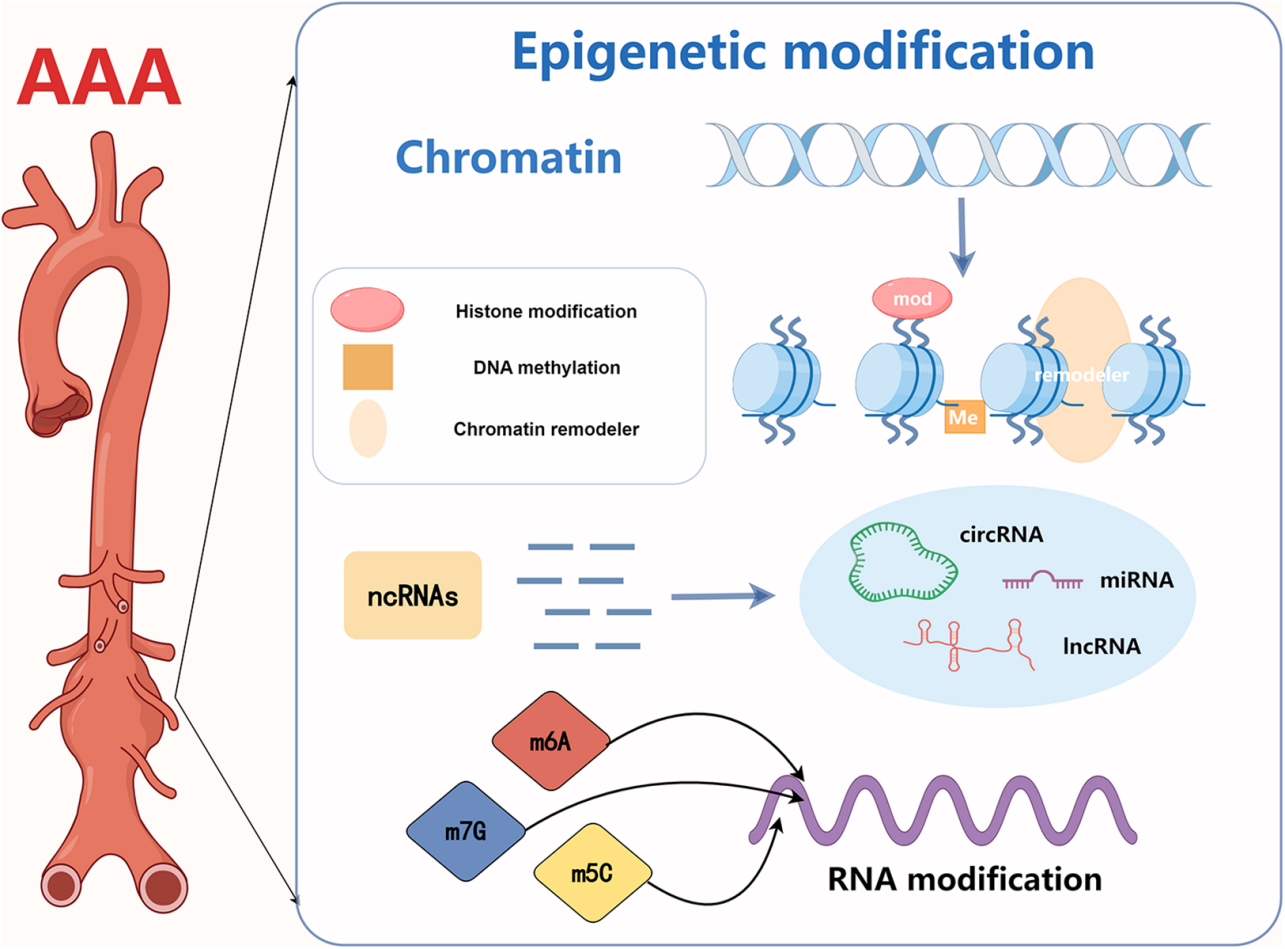
Abstract figure. Current research on epigenetics in AAA focuses on DNA methylation, histone modification, non-coding RNA action, and RNA modification.

This section focuses on the mechanisms of four epigenetic modifications in AAA, including DNA methylation, histone modification, non-coding RNA action, and RNA modification At present, DNA methylation, histone modification, non-coding RNA modification and RNA modifications, are commonly studied epigenetic mechanisms in AAA. The mechanisms have been briefly visualized in figure and detailed at the beginning of each part. However, epigenetic mechanisms also encompass other types that have not been extensively explored in AAA, and their roles in diseases are yet to be fully elucidated. Examples include chromatin remodeling and other mechanisms Figure 1.

### 2.1 DNA methylation in abdominal aortic aneurysms

**Figure 2:**
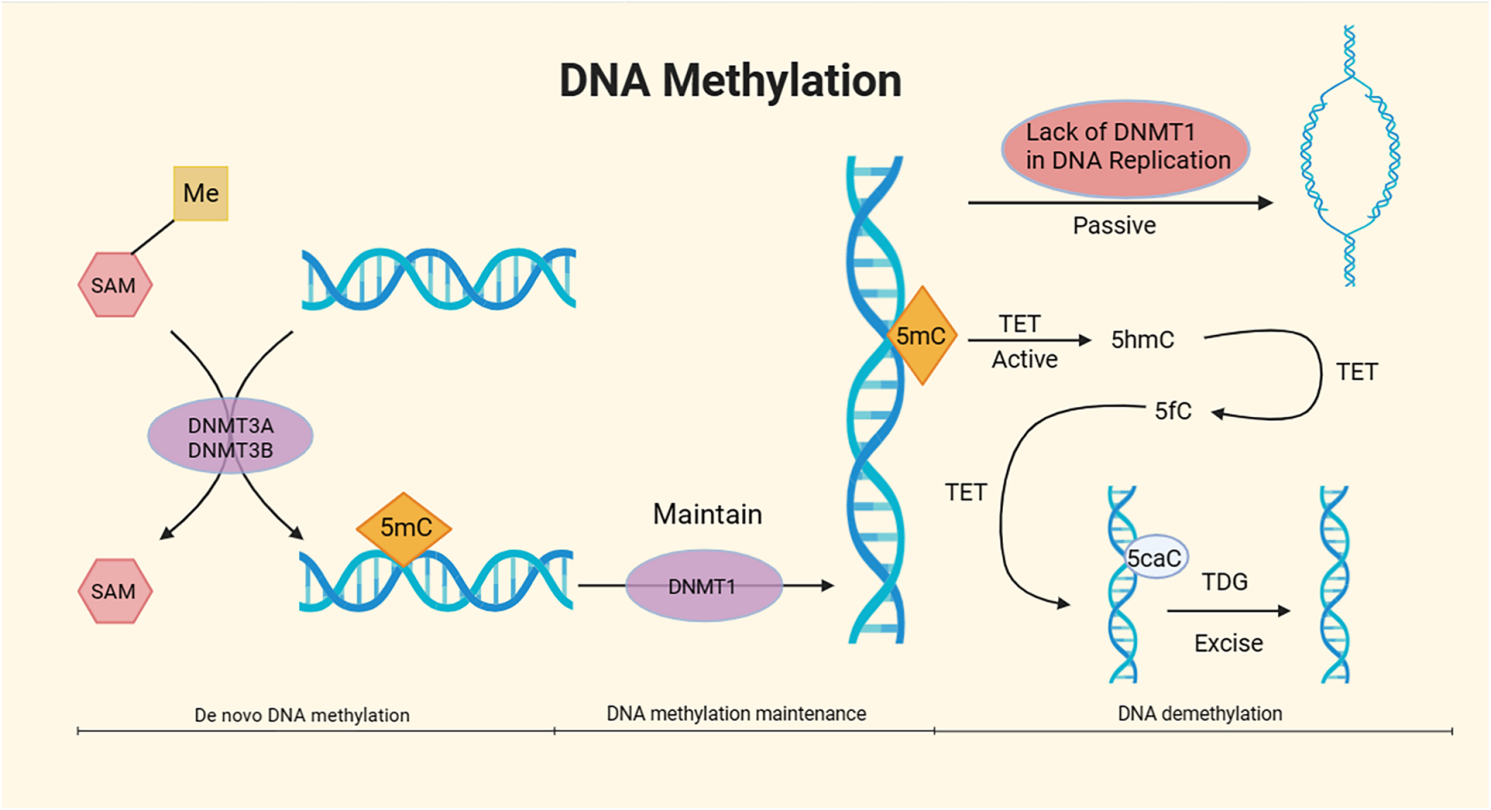
Mechanism of DNA methylation. DNA methylation, under the catalytic action of DNA methyltransferases (DNMTs) using S-adenosyl methionine (SAM) as the methyl donor, involves the covalent binding of a methyl group to specific DNA base positions, resulting in the formation of 5-methylcytosine (5mC) at the fifth carbon of cytosine. The overall process of DNA methylation is dynamic and can be divided into three stages: *de novo* DNA methylation, DNA methylation maintenance, and DNA demethylation. The DNMT3A and DNMT3B subtypes in DNMTs are responsible for *de novo* methylation of 5mC, while the DNMT1 subtype maintains methylation (24, 25). DNA demethylation mainly comprises passive demethylation and active demethylation. In the process of passive DNA demethylation, the absence of DNMT1 due to multiple DNA replications prevents the maintenance of DNA methylation (26). In active DNA demethylation, active DNA demethylation involves the Ten-Eleven translocation (TET) enzymes, which oxidize 5mC to 5-hydroxymethylcytosine (5hmC). TET further oxidizes 5hmC to 5-formylcytosine (5fC). Under the action of TET, 5fC is then oxidized to 5-carboxylcytosine (5caC), which is subsequently excised by thymine DNA glycosylase (TDG) in the base excision repair (BER) pathway (27, 28).

DNA methylation in cardiovascular diseases is gaining attention; however, its role in AAA has not been comprehensively studied. It is well known that smoking is a significant risk factor for AAA, and smoking has a crucial impact on the growth and rupture of AAA (21). As mentioned earlier, DNA methylation is controlled by DNMTs. Nicotine itself has been shown to not only affect the expression of DNMT1 but also influence promoter methylation levels in GABA-ergic neurons (22). Atherosclerosis is a complex pathological process involving vascular wall cells and inflammatory cells. As one of the risk factors for AAA, the mechanisms of atherosclerosis may be correlated with AAA. Endothelial dysfunction is the pathological basis of atherosclerosis and is accompanied by changes in vascular wall permeability. Its pathological process is closely related to abnormal DNA methylation (23). Other risk factors for AAA, such as hypertension and diabetes, may also involve similar mechanisms in AAA, but further correlational research is needed Figure 2.

Early studies have demonstrated a significant elevation in plasma homocysteine (Hcy) levels in AAA patients, and hyperhomocysteinemia (HHcy) is associated with the expansion rate of AAA (29). AAt the same time, a study's findings indicate that Hcy does not alter the protein levels of DNMT1 and DNMT3 but selectively reduces the activity of DNMT1 by 29% (30). Elevated Hcy levels may promote the development of AAA through multiple interrelated mechanisms, including endothelial dysfunction, protein hydrolysis, endoplasmic reticulum stress, enhanced inflammation, and increased cell apoptosis (31), which may indicate that HHcy in AAA may play a crucial role in AAA progression by reducing DNA methylation. Although research has shown an association between Hcy level and the growth rate of AAA, the conclusions drawn are relatively weak due to AAA being a multifactorial disease. However, a studies have indicated that patients with HHcy exhibit a faster expansion rate compared to those with normal Hcy levels, with a considerable number of patients demonstrating rapid expansion (>10 mm/year), thereby increasing the risk of rupture (32). Another study aimed at exploring the direct causal relationship between AAA and HHcy also suggests that HHcy may exacerbate AAA formation at least partially through the activation of peripheral fibroblast NADPH oxidase 4 (33). These two studies further confirm the high association of AAA with HHcy. A cohort study by Kristina Sundquist et al. provides an alternative perspective by confirming overall high methylation in AAA cases and concluding a significant correlation between overall DNA methylation, Hcy, and the baseline diameter of AAA (34). However, this study did not establish a correlation between overall DNA methylation and plasma Hcy levels. In the future, further studies may be carried out to further explore the specific mechanism of HHcy in promoting the growth and rupture of AAA by mediating DNA methylation.

Moreover, the diminished inhibitory effect of regulatory T cells (Tregs) may contribute to the progression of AAA, which is closely related to DNA methylation. Research suggests a reduced expression of FOXP3 (a transcriptional regulatory factor) in peripheral CD4CD25 Tregs of AAA patients, leading to functional deficiencies in overall CD4CD25 Tregs, indicating impaired immune regulation by Tregs, which may contribute to the disease progression of AAA (35). Another study confirms significantly higher DNA methylation levels in Tregs of AAA patients compared to healthy subjects, indicating a transcriptionally suppressed state affecting cellular functions (36). Furthermore, changes in DNA methylation have been shown to impact the expression of various genes related to vascular smooth muscle cell (VSMC) apoptosis, inflammation, and ECM degradation. This imbalance may be associated with the dysregulation of MMPs and TIMPs, closely linking them to AAA (17). These findings warrant further research for a comprehensive understanding of the mechanistic role of DNA methylation in AAA.

In addition, there have been some exciting advances in genome-wide DNA methylation. Evan J. Ryer et al. determined the genome-wide DNA methylation in peripheral blood mononuclear cells (PBMCs) of abdominal aortic aneurysm (AAA) patients, discovering significant differences in DNA methylation at specific CpG islands (CGIs). However, the observed differential methylation was noted to be potentially age-related rather than AAA-related (37). Additionally, the correlation between DNA methylation and gene silencing increases with the density of CpG dinucleotides at the promoter region (38). The human genome has fewer CpGs, but more CGIs. CGIs, relatively small 5′ end promoter regions, may experience gene suppression due to methylation of the fifth carbon of cytosine in this region (39). Aberrant CGI hypermethylation is common in tumor progression and may lead to abnormal gene silencing (40). Furthermore, the variations in 5mC, 5hmC, TET family proteins, and DNMTs at the genome-wide level differ depending on the type of cancer (24, 41) potentially serving as relevant interrogation sites for DNA methylation. Toghill BJ et al. used next-generation sequencing (NGS) on VSMCs collected from individual aortic tissues, pioneering the determination of CpG methylation status in regulatory regions of genes located at AAA risk loci identified in genome-wide association studies (GWAS) (42). With technological advancements, epigenome-wide association studies (EWAS) have evolved after GWAS, providing a systematic approach to revealing epigenetic variations underlying common diseases. EWAS, applied for a decade to analyze DNA methylation variations in complex diseases, has unveiled new molecular mechanisms for various common diseases, such as rheumatoid arthritis, metabolic syndrome, breast cancer, Alzheimer's disease, etc. It has made the application of epigenetic variations as biomarkers possible (43). This approach may lead to innovative research perspectives on the mechanisms of DNA methylation in AAA, offering molecular-level advancements in understanding the pathogenesis of AAA.

### 2.2 Histone modification in abdominal aortic aneurysms

**Figure 3:**
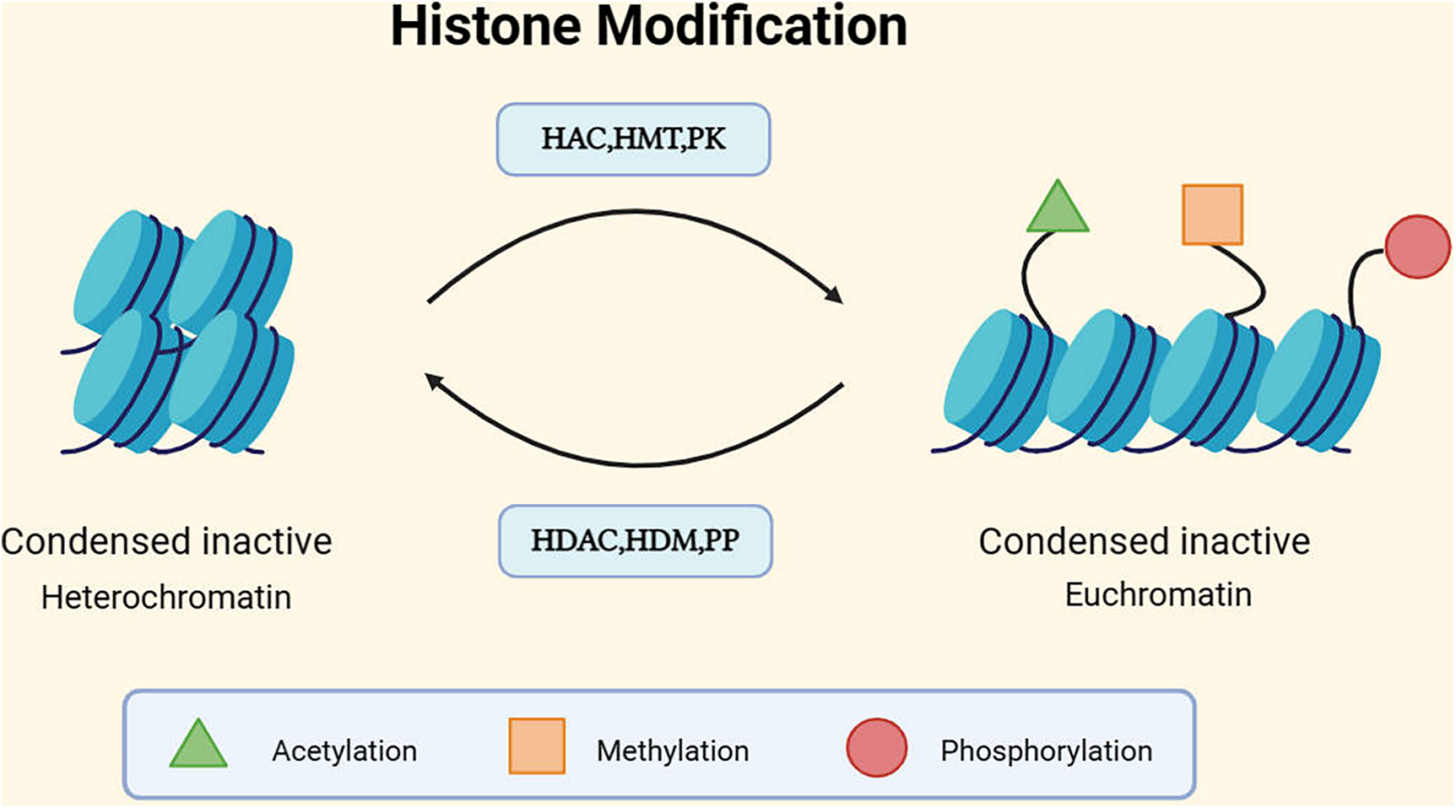
Mechanism of histone modification. DNA is wrapped around a histone octamer to form a nucleosome, and nucleosomes are the basic units of chromatin. The basic units of nucleosomes, after folding, constitute chromatin (49). Generally, there are two different forms of chromatin: euchromatin and heterochromatin, corresponding to the activation and silencing of gene expression, respectively. Euchromatin tends to have a more open conformation, supporting gene transcription, while the dense structure of heterochromatin tends to inhibit gene transcription (50). The structure of chromatin can be modulated through post-translational modifications (PTMs) of histone tails. Currently, the most studied histone covalent modifications include acetylation, methylation, and phosphorylation. In addition to these, there are ubiquitination, sumoylation, ADP-ribosylation, deimination, and proline isomerization, among others (51). Each modification has its corresponding enzymes that act on different histone residues. For instance, histone acetylation involves histone acetyltransferases (HATs), which are typically associated with the more open conformation of euchromatin. The action of histone deacetylases (HDACs) counteracts the effects of HATs and restores the positive charge of lysine side chains. HDAC/HAT constitutes a reversible enzyme pair that regulates histone acetylation PTM (52). Methylation is regulated by histone methyltransferases (HMTs) and demethylases (HDMs). Histone phosphorylation, similar to histone acetylation and methylation, is also a highly dynamic process, protein kinases (PKs) and phosphatases (PPs) respectively add and remove histone modifications, collectively controlling the overall level of modifications (53).

Research on histone acetylation and methylation modifications in abdominal aortic aneurysm (AAA) has been extensive. In the related studies of histone acetylation, Han et al. compared human AAA tissue with healthy aortic tissue, analyzing the differences in histone acetylation and the expression of corresponding HATs. The results showed significant overexpression of three lysine acetyltransferase (KAT) family members in the AAA vascular wall. Some lysine acetyltransferases, such as KAT2B, were found to be correlated with AAA diameter. High expression of KAT2B, KAT3B, and KAT6B was also observed in inflammatory cells (44). Another study found higher levels of H3 and H3K14 acetylation in T lymphocytes of AAA patients (45). However, contrasting results have been reported. As mentioned earlier, HDAC/HAT constitutes a reversible enzyme pair regulating histone acetylation PTM. Galán M et al. found that HDACs corresponding to KAT were significantly upregulated in AAA. In a mouse model, HDAC inhibitors were observed to restrict aneurysm progression. The study suggested that HDAC may promote processes such as ECM degradation, increased inflammatory mediators, and VSMC apoptosis (46). While some studies in mouse models have demonstrated inhibitory effects of HDAC inhibitors on MMP expression, the specific mechanisms require further investigation (47). Qian Xia et al. also identified a decrease in acetylation rates in H3 and H3K9, attributing it to the elevated influence of HDAC1 and HDAC5. HDAC1 and HDAC5 were found to inhibit the transcriptional activity of regulatory T cells (Tregs) (36). Additionally, Jacob Greenway et al. detected downregulation of H3K4me1, H3K9me3, and H3K56ac levels in two animal AAA models. The study revealed dynamic changes in histone H3 modifications during AAA formation, with no observed upregulation in H3 modifications (48). The discrepancies in results may be attributed to the levels of HAT and HDAC expression and their alterations during AAA development. Moreover, differences in modifications at specific sites vs. global modifications, as well as species variations, risk factors, and control tissue, need further in-depth research to address these disparities and determine the processes regulating histone methylation and acetylation patterns in AAA Figure 3.

In addition, there are novel findings regarding histone methylation. Research has revealed that histone demethylase JMJD3 stimulates the pro-inflammatory monocyte/macrophage phenotype in AAA tissue by selectively removing inhibitory histone H3K27me3 methylation. This leads to vascular remodeling and aortic dilation, promoting the progression of AAA (54). Another study reported that SETDB2, a histone methyltransferase, specifically trimethylates lysine 9 on histone H3 (H3K9me3), reducing the expression of inhibitory histones. This results in the loss of TIMP expression, causing dysregulation of MMP activity, aortic wall degeneration, and AAA formation (55). Numerous studies have demonstrated that, apart from histone acetylation, methylation, and phosphorylation, histone modifications also include ubiquitination and butyrylation. These modifications not only participate in the progression of metabolic diseases (56), but also impact the tumor microenvironment (57).

Furthermore, histone modifications, particularly histone methylation and acetylation, which have been extensively studied, play a regulatory role in cardiovascular diseases, including AAA risk factors such as atherosclerosis and hypertension (16). These study of histone modifications will play some inspiring roles in the study of the mechanism of AAA disease progression. In addition, histone modifications also include phosphorylation mentioned above and some novel modifications studied in cancer, such as lactatation, ubiquitination, lysine, citrullination, etc. These modifications may be closely related to metabolites (58). Exploring other histone modifications may provide new directions for studying the pathogenesis and treatment of AAA and requires further investigation. On this basis, since nucleosomes formed after DNA binding to histones control the structure of chromatin, the levels of DNA methylation and histone modification can also be expanded, and the microscopic to macroscopic study can be carried out at the level of chromatin remodeling, so as to further explore the disease progression of AAA.

### 2.3 Non-coding RNA in abdominal aortic aneurysms

**Figure 4:**
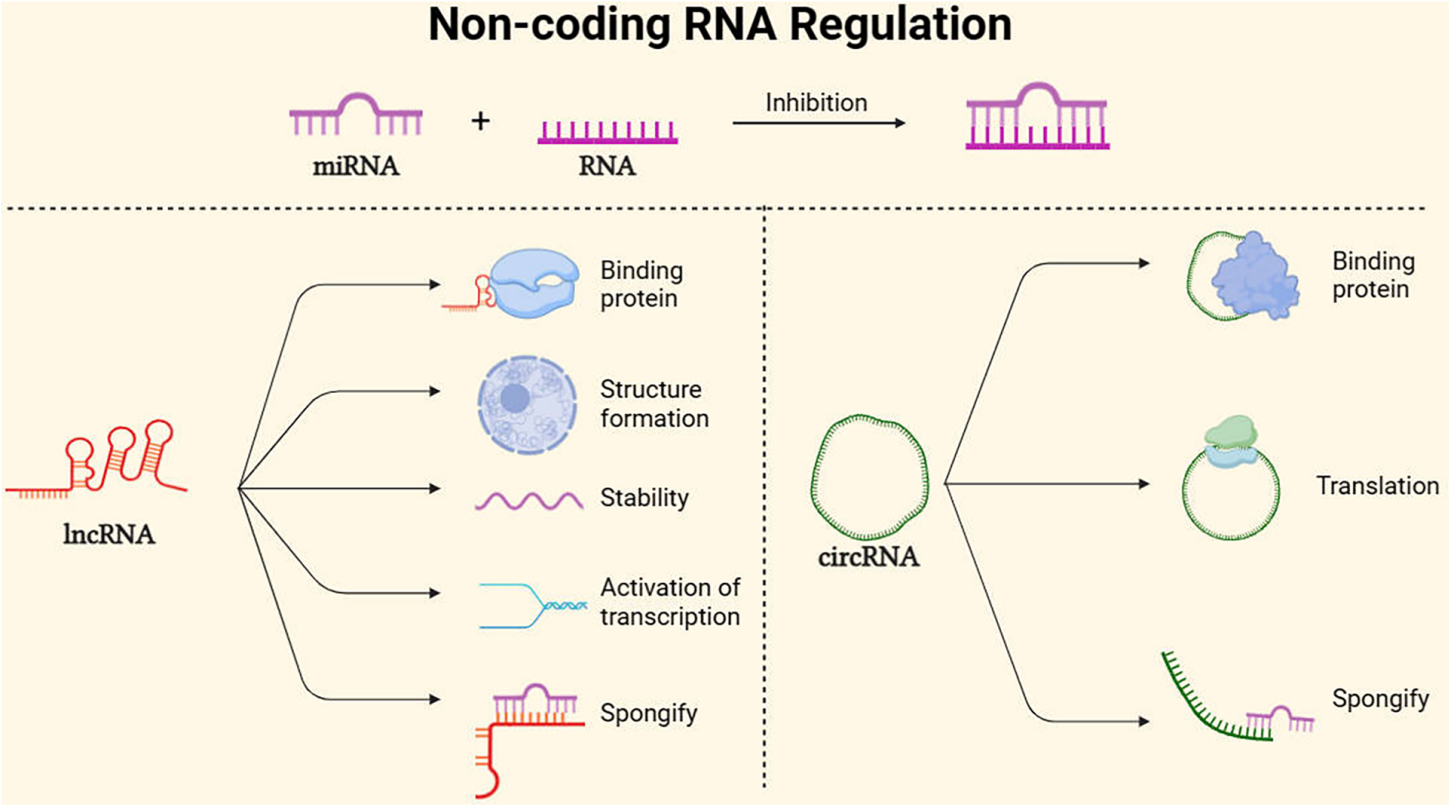
Main non-coding RNA regulation mode. MicroRNAs (miRNAs) are endogenous RNA molecules of approximately 23 nucleotides in length. They guide post-transcriptional expression inhibition of target genes by binding and pairing with the 3′ UTR of protein-coding RNA (mRNA) (60). Long non-coding RNAs (lncRNAs), with lengths exceeding 200 nucleotides and lacking protein-coding capacity, can act as scaffolds to regulate the expression of various genes, promote the formation of nuclear structures, and localize to specific DNA sites, In addition, lncRNAs can maintain mRNA stability, activate translation, sponging mRNA (61). Additionally, circular RNAs (circRNAs), Because of its stability, it can not only serve as a translation template for proteins, but also spongify competition to inhibit mirnas. At the same time, it can bind proteins to regulate gene expression pathways (62).

Approximately only 2% of the human genome encodes proteins, while non-coding RNAs (ncRNAs) are RNA molecules that do not encode proteins. In addition to their roles in transcription, ncRNAs may play complex and crucial epigenetic regulatory roles in higher animals. These ncRNAs, often referred to as regulatory RNAs, participate in biological processes such as gene expression regulation and chromatin structure modulation by interacting with DNA, RNA, and proteins (59). This section focuses on the above three ncRNAs that have been studied in AAA so far Figure 4.

#### 2.3.1 miRNA

The types and functions of miRNAs in AAA are diverse, and their regulatory roles vary. The following summary outlines the current understanding of miRNA regulation in AAA based on animal models and *in vitro* experiments. Some common target genes and regulatory functions may provide insights into the treatment of AAA.

#### 2.3.2 lncRNA

The main function of miRNA in gene regulation is to silence the expression of target genes by binding to mRNA. In contrast, the mechanism of action of lncRNA is more complex, as mentioned earlier, serving as a scaffold to regulate the expression of various genes, promote nuclear structure formation, and localize specific DNA sites, among other functions (61). Although there is limited research on lncRNA in AAA, studies have demonstrated its regulation in cardiovascular diseases (CVD) such as hypertension and atherosclerosis in vascular smooth muscle cells, involving various pathological and physiological changes (63).

H19 is a specific lncRNA associated with AAA. Li et al. established two mouse models of AAA and analyzed the RNA transcription expression in the models. The results indicated that the upregulation of H19 is correlated with the progression of AAA, including the content and apoptosis of SMCs. *In vitro* experiments demonstrated a positive correlation between H19 expression and the apoptosis rate of human aortic smooth muscle cells, suggesting that lncRNA H19 is a novel regulatory factor in the initiation and development of AAA (64). Another study demonstrated the role of H19 in enhancing vascular inflammation and inducing AAA formation. H19 was found to enhance vascular pro-inflammatory IL-6 and MCP-1 and promote AAA formation by enhancing macrophage infiltration (65). X-inactive specific transcript (XIST) is an lncRNA located on the X chromosome, and its role in certain cancers has been elucidated, including breast cancer, rectal cancer, oral cancer (66–68). XIST regulates apoptosis and proliferation through competitive miRNA mechanisms, closely related to the development and growth of tumors (69, 70). Research has also shown that XIST in thoracic aortic aneurysm (TAA) acts as a “sponge” to absorb miR-29b-3p, leading to overexpression of elastin (Eln) in VSMCs and promoting smooth muscle cell apoptosis, indicating its role in aneurysm development (71). AAA also involves abnormal proliferation and apoptosis of VSMCs. Corresponding research results are similar to those in TAA, mainly related to competitive miRNA pathways. Studies have shown that upregulation of XIST can competitively interact with miR-1264 in the WNT/β-catenin signaling pathway and competitively interact with miR-762-mediated mitogen-activated protein kinase kinase 4 (MAP2K4) pathway, thereby inhibiting apoptosis and promoting the proliferation of VSMCs, contributing to the development of AAA (72, 73).

Many recent studies have highlighted the involvement of various lncRNAs in AAA. For instance, lncRNA PVT1 promotes VSMC apoptosis, ECM degradation, and pro-inflammatory factor production by upregulating MMP-9. On the other hand, lncRNA PVT1 acts as a sponge for miR-3127-5p/NCKAP1l, inhibiting VSMC proliferation, inducing apoptosis, and activating inflammation, thus promoting AAA progression (74, 75). Upregulation of lncRNA NEAT1 accelerates VSMC proliferation and inhibits replicative cell apoptosis through the miR-4688/TULP3 pathway (76). The interaction between lncRNA TUG1 and transcriptional repressor KLF4 mediates impaired SMC differentiation function (77). Elevated lncRNA Sox2ot enhances oxidative stress and inflammation in VSMCs through competitive regulation of the miR-145/Egr1 pathway (78) GAS5 overexpression inhibits cell proliferation, induces SMC apoptosis, and accelerates AAA formation in a mouse model. GAS5 acts as a sponge for miR-21, inhibiting the miR-21/PTEN pathway and Akt phosphorylation. Additionally, GAS5 forms a positive feedback loop with Y-box binding protein 1 (YBX1), promoting downstream p21 expression, inhibiting SMC proliferation, and inducing apoptosis (79), GAS5 also induces SMC apoptosis through activation of the EZH2-mediated RIG-I signaling pathway (80). NUDT6, a conservative antisense transcript of FGF2, impairs SMC migration, limits proliferation, and enhances apoptosis, promoting AAA and carotid artery diseases (81). LINC00473 regulates the miR-212-5p/BASP1 pathway to exert anti-proliferative and pro-apoptotic effects in VSMCs (82). Overexpression of CRNDE upregulates Smad3 via Bcl-3, promoting VSMC proliferation and inhibiting cell apoptosis in AAA (83). SENCR overexpression may inhibit AAA formation by suppressing VSMC apoptosis and extracellular matrix degradation, but the specific pathway mechanism needs further exploration (84). These studies provide some scientific basis for understanding the pathogenesis and potential treatments of AAA involving lncRNAs. While many of these lncRNAs act on corresponding miRNAs, exploring other mechanisms of lncRNA epigenetic regulation might yield unexpected therapeutic effects in AAA treatment. Additionally, the clinical translation of these findings requires further investigation. The progression of AAA is a long-term process involving different epigenetic changes at different stages. Therefore, clinical research with sufficient sample sizes and excellent staging and indicator selection is crucial for precise and feasible AAA treatment.

#### 2.3.3 CircRNA

CircRNAs, as a relatively novel research avenue in AAA, primarily exert their effects through miRNA sponging, possibly achieved through competitive endogenous RNA (ceRNA) mechanisms. Bioinformatics studies have explored potential molecular mechanisms in AAA by constructing ceRNA interaction networks involving circRNAs and miRNAs (85). For instance, circCBFB acts as a miR-28-5p sponge, promoting the reduction of GRIA4 and LYPD3 levels, thereby decreasing VSMC apoptosis and facilitating AAA progression (86). Novel circRNAs such as circ_0092291 might inhibit Ang II-induced smooth muscle cell damage by sponging miR-626, leading to increased levels of collagen IV alpha 1 chain (COL4A1) and suppressing AAA development (87). Circ_0002168 regulates VSMC proliferation and apoptosis by sponging miR-545-3p to enhance CKAP4 expression, potentially impacting VSMC loss in AAA (88). Another circRNA, hsa_circ_0087352, upregulated in AAA, may enhance macrophage inflammation and regulate VSMC apoptosis by sponging has-miR-149-5p and acting on LPS (89). Furthermore, circRNAs can influence protein pathways. For instance, circChordc1 induces wave protein degradation, increases GSK3β/β-catenin pathway activity, promotes VSMC phenotype transition, reduces apoptosis, and alleviates vascular remodeling to inhibit AAA progression (90). CircCdyl promotes vascular inflammation and induces M1 polarization in macrophages, contributing to AAA formation, by inhibiting interferon regulatory factor 4 (IRF4) nuclear entry and acting as a sponge for let-7c to enhance C/EBP-δ expression (91). Additionally, circRNA transcription, such as that of the circRNA of the ataxia-telangiectasia mutated gene (cATM), may represent an early characteristic change in the AAA microenvironment, triggering oxidative stress reactions in SMCs and potentially serving as a crucial molecular diagnostic indicator for AAA (92). Despite these findings, research on the role of circRNAs in AAA remains incomplete. Mechanisms involving other miRNA sponging, as well as alternative roles of circRNAs, such as protein translocation, translation, and interactions between proteins, require further exploration in subsequent studies.

Certainly, the involvement of ncRNA and epigenetic regulation extends beyond the mentioned three types. For instance, pathways related to piwi-interacting RNA (piRNA) and the target sites of small interfering RNA (siRNA) also contribute. Notably, studies have indicated the potential involvement of the piRNA pathway, specifically piRNA piRPG, in AAA (93). Further in-depth research is warranted to explore the specific roles of other ncRNAs in AAA pathogenesis.

Due to the complex and multifactorial nature of AAA, ncRNA may represent a potential factor for effective treatment. This is because ncRNAs can regulate the expression of corresponding proteins by targeting multiple mRNAs and influencing various signaling pathways related to AAA. NcRNA is currently in the exploratory stage, and when transcribing multiple genes, only a portion of them may be translated into proteins. Other genes are intricately regulated by a network of non-coding RNAs, controlling the expression of the final protein products. Numerous studies emphasize the significance of ncRNAs, as they play critical roles in the development of diseases. Moreover, these research findings are actively translating into clinical therapies. NcRNAs may serve as the foundation for clinical targeted treatments, and their role in screening and monitoring early stages of the disease is crucial for making accurate therapeutic decisions (94).

### 2.4 RNA modification in abdominal aortic aneurysms

In recent years, an increasing body of research has highlighted the crucial role of epigenetic factors in the onset and progression of cardiovascular diseases (95–98). RNA methylation is a significant form of epigenetic modification, belonging to the fields of epitranscriptomics and epigenomics, which collectively regulate gene expression in eukaryotes (99, 100), this includes 6-methyladenosine (m6A), 5-methylcytosine (m5C), and N-7-methylguanosine (m7G). Currently, studies regarding the association between RNA methylation and AAA are limited, with the primary focus on m6A.

#### 2.4.1 m6A modification

m6A modification involves methylation of the sixth nitrogen atom on the adenine base of RNA, and this modification is widely present in eukaryotes, playing a role in the regulation of certain non-coding RNA metabolism (101). The enzymes associated with m6A modification are categorized into methyltransferases (writers), demethylases (erasers), and m6A-binding proteins (readers), forming a crucial protein ensemble that can add, remove, and recognize m6A modification sites, thereby altering biological processes (102). Major methyltransferases include METTL3, METTL14, WTAP, RBM15/15B, KIAA1429, ZC3H13, and METTL16, while demethylases comprise FTO and ALKBH5. Recognized readers include YTHDF1, YTHDF2, YTHDF3, YTHDC1, YTHDC2, HNRNPC, HNRNPG, among others (103). m6A methylation profoundly impacts various aspects of RNA metabolism, including RNA expression, splicing, translation, and RNA-protein interactions (104). It participates in the regulation of multiple biological processes, such as autophagy, inflammation, oxidative stress, DNA damage, and cellular aging (105–107), all of which are closely associated with the occurrence and progression of AAA. AAA is characterized by vascular remodeling and progressive dilation (1), infiltration of lymphocytes and macrophages into the vessel wall, and smooth muscle cell apoptosis (108). Studies have revealed a significant increase in m6A modification levels in AAA, with elevated m6A posing an increased risk of AAA rupture (109, 110). Moreover, differential expression of METTL14, FTO, and YTHDF3 has been observed in various types of inflammatory cells in AAA tissue. FTO, in particular, has shown a strong correlation with the infiltration of aneurysmal smooth muscle cells and macrophages, indicating its potential pivotal role in AAA progression (111). Furthermore, METTL14, HNRNPC, and RBM15 exhibit significant expression differences in AAA and are strongly associated with infiltrative immune cells such as macrophages and mast cells (112). RBM15, by recruiting the WTAP-METTL3-METTL14 RNA methyltransferase complex, enhances m6A levels. Knocking down RBM15 can reduce the expression of m6A-dependent CASP3, inhibiting apoptosis in human abdominal aortic smooth muscle cells (113, 114). In addition, a study by Zhong et al. found that METTL3/m6A participates in AAA formation by promoting the expression of mature miR34a, thereby reducing the expression of SIRP1, providing new targets and diagnostic biomarkers for clinical treatment of AAA (115). Current research on the mechanism of m6A action in AAA mostly focuses on FTO, METTL3, and METTL14, and further investigation is needed to explore the interactions between other regulatory factors.

#### 2.4.2 m5C modification

m5C modification refers to the methylation of the fifth carbon atom on the cytosine base of RNA molecules, a process predominantly catalyzed by enzymes of the NSUN family in eukaryotes. This modification participates in various RNA biological processes, including RNA export, translation, and ribosome assembly (116). Post-transcriptional m5C modification has been confirmed to play a crucial role in various cancer diseases such as lung cancer (117), prostate cancer (118), breast cancer (119), bladder cancer (120) among others. Currently, there is limited research on m5C modification in AAA. Several studies have indicated that NSUN2, by regulating mRNA stability and the translation process, influences a range of pathological processes, including cell proliferation, oxidative stress, and inflammatory responses (121–123). Furthermore, research has shown a significant increase in mRNA m5C modification levels in AAA compared to healthy groups (124). These findings suggest that m5C modification may play a role in the clinical mechanisms affecting AAA, providing new avenues for research in AAA treatment.

#### 2.4.3 m7G modification

m7G modification is one of the most common post-transcriptional base modifications, widely distributed in tRNA, rRNA, and the 5′ cap region of eukaryotic mRNA (125). The identified methyltransferases responsible for m7G methylation include the yeast Trm82/Trm8 complex and its mammalian homolog METTL1/WDR4 complex (126). To date, numerous research findings suggest that m7G modification influences the occurrence and development of various cancer diseases, such as hepatocellular carcinoma (127), esophageal cancer (128), and bladder cancer (129). Additionally, reports have indicated the significant role of m7G modification in inflammation and angiogenesis (130). It can be inferred that m7G modification may also impact the development of AAA. However, the specific mechanisms through which m7G modification affects AAA remain unclear. Bioinformatic analysis has revealed a significant correlation between AAA and m7G-related genes, including CYFIP1, EIF3D, EIF4E3, NSUN2, and NUDT11 (131). Nevertheless, further *in vivo* and *in vitro* experiments are needed to validate the mechanisms through which m7G modification influences abdominal aortic aneurysm.

RNA modifications extend beyond the mentioned types. For example, 3-methylcytidine (m3C) is close to m5C sites, and 7-methylguanosine cap structure [m7Gpp(pN)] acts on dihydrouridine (D) and pseudouridine (Ψ) in tRNA and rRNA. Research on these modifications has been conducted in breast cancer, gastric cancer, liver cancer, and prostate cancer, but investigations into the relationship between RNA modifications and AAA are still ongoing (132). Studies have shown that N1-methyladenosine (m1A) may promote macrophage polarization in aortic inflammation through the reader YTHDF3, affecting target gene expression and influencing the progression of AAA (133). Further research is needed to explore the specific mechanisms and roles of RNA modifications, paving the way for understanding the pathogenesis and treatment of AAA.

## 3 Epigenetic regulatory genes potentially associated with abdominal aortic aneurysm

In the context of epigenetic pathways related to AAA, some novel examples have caught our attention. They may be associated with the progression of AAA and could potentially regulate the expression of corresponding proteins through epigenetic mechanisms. The following outlines three examples related to AAA.

### 3.1 PCSKs

The PCSK family consists of nine proteases, with PCSK9 being demonstrated as crucial in the regulation of CVD (134). In a 2023 study, a potential therapeutic target, Proprotein Convertase Subtilisin/Kexin Type 9 (PCSK9), was identified through AAA's genome-wide association meta-analysis of 121 independent risk loci. When elastase was introduced into PCSK9 mice, AAA growth decreased, indicating a unique role for PCSK9 in AAA (135). Besides, Inhibitors of PCSK9 can reduce low-density lipoprotein (LDL) cholesterol levels, potentially decreasing the risk of severe cardiovascular disease symptoms associated with atherosclerosis (136). A large-scale study involving 100,000 individuals suggested that elevated lipoprotein levels may increase the risk of AAA 2–3 times (137). Given PCSK's role in regulating lipid metabolism, this represents the feasibility of PCSK as a therapeutic target, necessitating more preclinical research and clinical trials to assess its efficacy in AAA-related outcomes.

Meanwhile, epigenetic mechanisms likely play a role in some functional aspects of PCSK genes. For example, sirt6 deacetylase can inhibit PCSK9 gene expression by enriching the transcription factor Foxo3 in the proximal promoter region of the PCSK9 gene, leading to histone H3 deacetylation (138). Increased expression of has-miR-335 and has-miR-6825 can lower PCSK9 mRNA expression in myocardial cells (139). Additionally, the histone acetyltransferase P300 may increase PCSK9 expression (140). Non-coding RNAs (ncRNA) play a crucial role in the progression of inflammation in atherosclerosis by targeting genes related to the PCSK9 pathway at the post-transcriptional level (141). Further research is needed to uncover the pathway-related mechanisms of PCSK9 in AAA, potentially providing new insights and breakthroughs into the complex pathogenesis of AAA.

While PCSK9's Epigenetic importance is established, the specific mechanism of other PCSK family protein in AAA are still under exploration. Members of the PCSK family share structural similarities, but their functional roles vary. In cancer research, other PCSK family members have been reported to activate cytokines, influencing cell proliferation, migration, and extracellular matrix remodeling—important features in the progression of AAA (142). A study categorized PCSK analysis into plaque expression, biochemical blood parameters, morphological characteristics, and patient symptoms. The cumulative data from each section suggested the feasibility of PCSK6 as a new therapeutic target, followed by PCSK5, PCSK7, and FURIN, all showing potential for CVD treatment within this family (143). This, of course, is also closely related to the treatment of AAA. Therefore, the research on PCSK family proteins may be more in-depth and comprehensive.

### 3.2 TGF-β

The transforming growth factor-beta (TGF-β) family members include TGF-beta, pathway factors, and bone morphogenetic proteins (BMP), among other genes, with extensive research in cancer. TGFβs can activate various pathways, including the Sma- and Mad-related family proteins (Smad), mitogen-activated protein kinase (MAPK), and phosphoinositide 3-kinase (PI3K) pathways. Their association with AAA may lie in their ability to ultimately inhibit inflammatory cell infiltration, reduce ECM degradation, limit VSMC apoptosis, promote ECM formation, and be involved in tissue repair, fibrosis, extracellular matrix remodeling, cell proliferation, and migration (144, 145). It's worth mentioning that the Smad pathway, in terms of epigenetics, is regulated by various miRNAs during the AAA process. Notably, miRNA-29b-mediated Smad targeting facilitates AAA by influencing key matrix metalloproteinases (MMP-2 and MMP-9). Inhibiting miRNA-26a increases gene expression of SMAD-1 and SMAD-4, promoting vascular smooth muscle cell proliferation, inhibiting cell differentiation and apoptosis, altering TGF-β pathway signaling. Additionally, miR-424/322 analogs modulate Smad2/3/runt (146–149). Moreover, miRNA-195, miRNA-155, and miR-143/145 intersect with the Smad pathway in the TGF-β pathway (150). A genetic association study in a Dutch population analyzed single nucleotide polymorphisms (SNPs) in TGF-β receptor genes TGFBR1 and TGFBR2, revealing potential correlations similar to TAA in AAA (151). This may be evidence that some TGF-β is related to genetics or epigenetics. In the TGF-β pathway, multiple genes coordinate final outcomes, and aside from the Smad pathway, the other two pathways may form a complex and mutually influencing regulatory system with various non-coding RNAs. Furthermore, there is limited research on histone modification and DNA methylation in this context, necessitating further exploration of the pathways and discovery of new targets.

### 3.3 RTKs

Receptor tyrosine kinases (RTKs) form the largest class of enzyme-linked receptors, serving both as receptors for growth factors and as enzymes that catalyze downstream target protein phosphorylation. RTKs play crucial regulatory roles not only in normal cells but also have pivotal functions in the progression of various cancers, including lung cancer, gastric cancer, thyroid cancer, etc (152). Mutations in receptor tyrosine kinases lead to the activation of a series of signal cascades, impacting protein expression, including epigenetic effects, although specific research in this regard is currently lacking. RTKs can modulate various downstream signaling pathways such as MAPK, PI3K/Akt, and JAK/STAT. Various types of RTKs may be overexpressed in AAA, including epidermal growth factor receptors (EGFRs) (153), vascular endothelial growth factor receptors (VEGFRs) (154), platelet-derived growth factor receptors (PDGFRs) (155), insulin-like growth factor receptors (IGFRs) (156) and fibroblast growth factor receptors (FGFRs) (157). Research indicates that caspase recruitment domain and membraneassociated guanylate kinaselike domain protein 3 (CARMA3) recruits two downstream signaling molecules, BCL10 and MALT1, through its N-terminal effector CARD domain, forming the CARD11-BCL10-MALT1 (CBM) complex (158), involved in the NF-kB signaling pathway induced by G protein-coupled receptor (GPCR) and RTK. Inflammation is a critical feature in the pathogenesis of cardiovascular diseases, including Abdominal Aortic Aneurysm (159). During the inflammatory process, blood vessels and the surrounding connective tissue are important regulatory factors (160). In endothelial cells of mice, CARMA3 assembles BCL10 and MALT1 to form the CBM signalosome, triggering NF-kB activation induced by CXCL8/IL8. CXCL8/IL8 is a crucial chemokine involved in promoting angiogenesis and inflammation. This process controls VEGF expression and promotes autocrine activation of VEGF receptors (161). The CBM complex also mediates coagulation-induced NF activation (162). However, how CARMA3 connects to GPCR and regulates the NF-kB signaling pathway remains a puzzle. Future studies can explore how RTKs, through epigenetic mechanisms, influence NF-kB to impact the occurrence and development of AAA, thereby identifying new therapeutic targets.

The three aforementioned gene pathways represent only a minute fraction of the vast gene regulatory network. In addition to the previously discussed pathways, such as the inflammatory response pathways, including the Interleukin-6 (IL-6) pathway, and the MMP pathway responsible for degrading cellular tissue matrix, there is limited research on the interplay with epigenetics. Besides refining the epigenetic aspects of these pathways, further exploration and research are needed on gene pathways related to cellular autophagy, ECM, and VSMC proliferation, and their associations with epigenetic modifications.

## 4 Current drug and prospect of epigenetic treatment for abdominal aortic aneurysms

**Figure 5:**
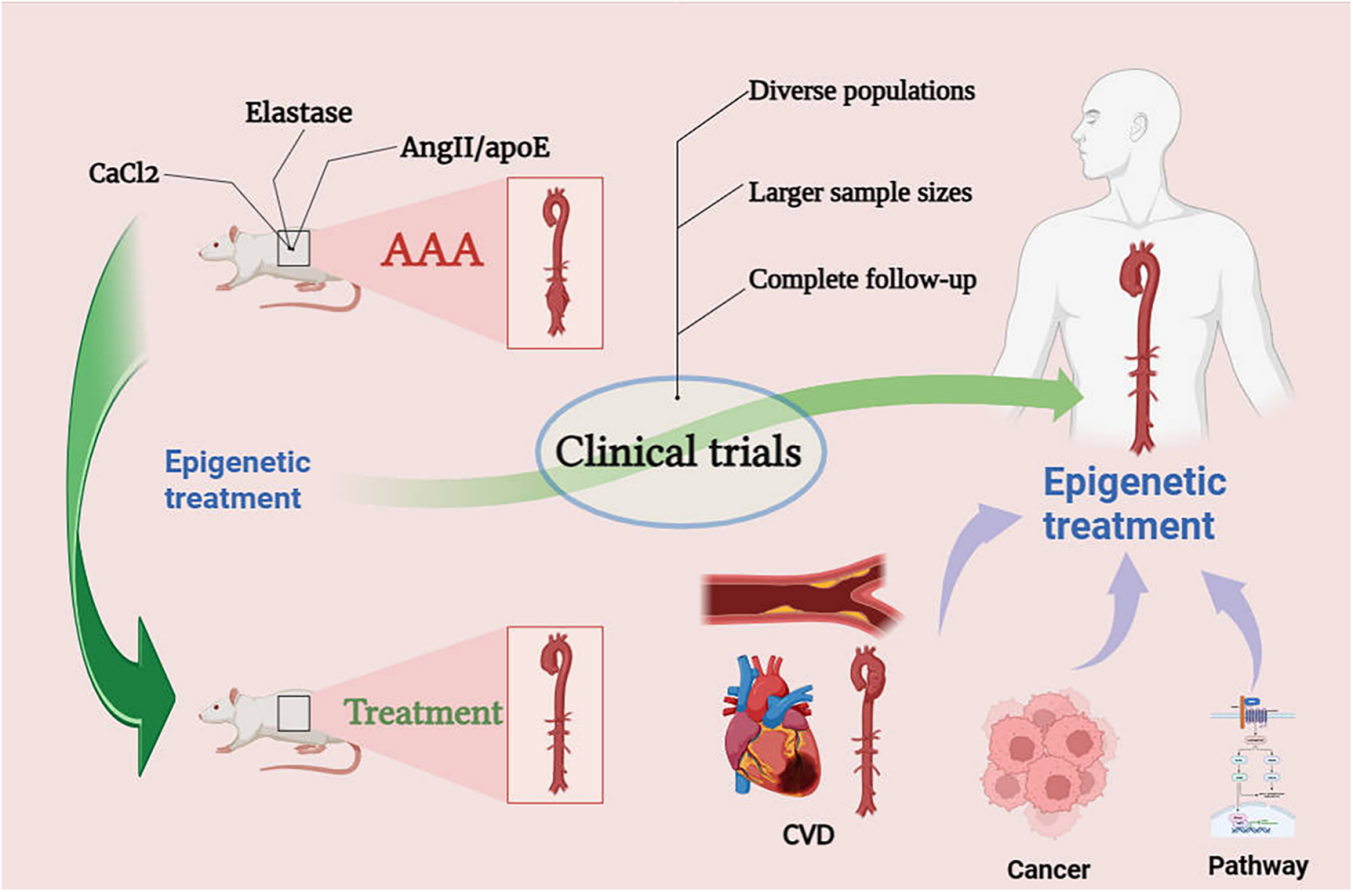
Prospects for epigenetic treatment of AAA. Epigenetic drugs need to go through clinical trials, we may be able to look for therapeutic ideas for AAA through epigenetics in the therapeutic mechanisms of CVD, cancer and pathways.

In preclinical studies of abdominal aortic aneurysm (AAA), three main rodent models are commonly employed: the elastase model, the CaCl2 model, and AngII/ApoE deficient mouse model. The elastase model emphasizes the role of inflammation-related mechanisms in AAA but may ultimately lead to healing unrelated to AAA rupture. The CaCl2 model can induce a smaller degree of aneurysm and, similar to the elastase model, does not result in AAA rupture. The AngII/ApoE deficient mouse model is the most commonly used, causing rupture but potentially having a higher correlation with aortic dissection, and its results may not necessarily translate to human AAA outcomes (163). As a matter of fact, a challenge in current experiments related to abdominal aortic aneurysm lies in the difficulty of translating some of the existing animal model results into clinical trial outcomes. On the one hand, animal models need continued optimization due to the physiological and biochemical differences between rodents and mammals. On the other hand, inherent differences exist between animal models and humans in various aspects. The pathogenesis of human AAA is highly complex, requiring interdisciplinary scientific approaches to prove the efficacy of drug treatments in the progression from small to large AAA after the discovery of small AAAs Figure 5.

### 4.1 Current drug for AAA

Due to studies demonstrating a common coexistence and positive correlation between Chlamydia pneumoniae infection and AAA progression (164, 165), antibiotics were once considered as potential candidates for clinical treatment. However, results from two large clinical studies showed that doxycycline treatment did not reduce aneurysm growth, nor did it delay the need for AAA surgery or the timing of surgical repair (166, 167). The most commonly used AAA animal model involves inducing abdominal aortic aneurysm formation with angiotensin II, and clinical trials are often conducted using ACE inhibitors (ACEIs) and angiotensin II receptor blockers (ARBs). Additionally, there are numerous clinical trials for anti-inflammatory drugs targeting immune cell inflammatory infiltration in AAA, statin drugs selected from observational experiments for their potential correlation with AAA growth and rupture, and antiplatelet drugs that weaken blood clots. It is noteworthy that metformin, targeting the risk factor of diabetes for AAA, is still under clinical trial. However, AAA growth is a long-term process influenced by multiple factors, including statistical considerations. Since age is also a risk factor for AAA, elderly AAA patients often end up with the outcome of loss to follow-up due to death. As a result, the statistical results of clinical trials are often censored, making them less convincing (9, 168, 169). Currently, most drug clinical trials involve repurposing basic drugs from other diseases. These trials test the impact of drugs on limiting AAA growth, but there is still no clinical trial data providing conclusive evidence of drug efficacy in restricting AAA progression. Further research may require larger sample sizes, diverse populations, and clinical trial designs that account for gender differences. Future exploration may also involve personalized targeting of drug development for AAA to translate the effects of AAA-related drugs into therapeutic outcomes.

### 4.2 Possibility of epigenetic modification in the treatment of AAA

Since the cellular aberrations observed in abdominal aortic aneurysms (AAA) involving proliferation, apoptosis regulation, inflammatory infiltration, and fibrosis are similar to those observed in cancer, the therapeutic significance of epigenetic modification in cancer may be instructive for AAA. It is plausible that similar treatment approaches could be applied. For example, Brd4, as an epigenetic regulator of multiple programmed cell death, has some untapped potential in cancer. This may indicate that it may serve as a potential direction for epigenetic therapy of AAA (170). In addition, m6A in RNA modification may play some crucial roles in tumor glycolysis, and the glycolytic reprogramming of cancer cells is a major characteristic of cancer, which may become a special therapeutic mechanism affecting the occurrence and development of tumors. It may represent that AAA may also have similar epigenetic mechanisms related to proliferation to be further studied (171). Immunological treatments targeting cell-specific epigenetic modifiers such as histone demethylase JMJD3 and SET domain bifurcated protein lysine methyltransferase 2 (SETDB2) have shown promise in effectively intervening in AAA progression (54, 55).

Exploring well-established pathways in cancer research for potential treatment targets is anotheravenue. For instance, the Wnt/β-catenin pathway has been found to be significantly activated in human and experimental animal AAA models, suggesting the presence of potential therapeutic targets, despite the exclusion of specific anti-tumor drugs for arterial disease in animal models (172). Due to the intricate genetic actions of pathways, in-depth research is necessary to determine whether improving arterial aneurysm development is achievable by targeting specific components of these pathways. The presence of ncRNAs may fulfill this role. Recent studies on lncRNA have shown that inhibiting H19 expression could serve as a novel molecular therapeutic target, preventing or slowing AAA progression by suppressing IL-6 expression (65). The role of miRNAs in AAA treatment has been demonstrated (Table 1), but a combination with specific pathways and target gene functions is still required. It is essential to explore the intersections of various pathways and ncRNAs in detail to identify critical therapeutic targets for AAA progression.

**Table 1:** The Role of miRNA in AAA.

The miRNA in AAA					
miRNA types	Up-regulated (++)/Down-regulated (−)	Gene	miRNA regulatory function	Sample	Reference
miR-1-3p	–	TLR4	Inducing the formation of dedifferentiated cell phenotypes and inflammation	hASMC	(173)
miR-10b	+	CMA1, LCN2	Promotion of degradation of elastin, elevation of neutrophil and mast cell markers	Mouse whole aorta	(174)
miR-15a-5p	+	CDKN2B	Inhibition of apoptosis of SMCs	hASMC	(175)
miR-17-5p	−	TXNIP, NLRP3	Inhibition of inflammatory cytokine release, reducing macrophage pyroptosis	Mouse ADSC	(176)
miR-19b-3p	−	MST4	Inhibition of stem cell proliferation, suppression of VSMC senescence	Mouse whole aorta	(177)
miR-21	+	PTEN	Promotion of aortic wall cell proliferation and reduction in cell apoptosis	Mouse whole aorta, hASMC, hAFB 和 hAEC	(178)
miR-23b	−	FoxO4	Inhibition of VSMC phenotype switching	Human AAA tissue and mouse aortic VSMC	(179)
miR-24	−	CHI3L1	Promotion of inflammation in macrophages, SMCs, and VECs	hASMC, hAEC, mouse whole aorta	(180)
miR-26a	−	Smad1, Smad4	Promotion of vascular SMC proliferation while concurrently inhibiting cell differentiation and apoptosis	hASMC	(146)
miR-28-5p	+	GRIA4, LYPD3	Promotion of SMCs apoptosis	hASMC	(86)
miR-29a-3p	+	PTEN	Promotion of AECs proliferation	hAEC, mouse AEC	(181)
miR-29b	−	Col1a1, Col3a1, Col5a1, Eln	Inhibition of perivascular fibrosis and decreasing collagen content	hASMC, hAFB	(147)
miR-126	−	ADMA9	Promotion of macrophage infiltration, cell apoptosis, and inflammation	hASMC, hAoECs	(182)
miR-126-5p	−	VEPH1	SMCs synthesis and phenotype transition	hASMC	(183)
miR-126a-5p	−	ADAMTS-4	Inhibition of the infiltration of inflammatory macrophages	hASMC	(184)
miR-144-3p	+	EZH2	Promotion of SMCs proliferation and inhibit cell apoptosis	hASMC	(185)
miR-144-5p	−	TLR2 and OLR1	Inhibition of the M1 macrophage polarization	Macrophages	(186)
miR-147	+	EV	Promotion of the macrophage inflammatory response	hAMSC	(187)
miR-155-5p	+	FOS, ZIC3	Inhibition of the activity of VSMCs and reduce cell apoptosis	hASMC	(188)
miR-155	+	MMP-2, MMP-9, iNOS, MCP-1	Promotion of the proliferation and migration of VSMCs	hVSMC	(189)
miR-181b	+	TIMP-3	Inhibition of the expression of elastin and collagen proteins	Human and Mouse Abdominal Aortic Macrophages	(190)
miR-194	−	KDM3A	Inhibition of inflammatory response and oxidative stress	Mouse VSMC	(191)
miR-195	+	Smad3	Inhibition of proliferation, induce cell apoptosis, and increase the expression of collagen II and OPN	hASMC	(192)
miR-199a-5p	+	Sirt1	Promotion of ROS generation and VSMC senescence	hASMC	(193)
miR-205	+	LRP1	Impede the removal of MMP-9 from the cellular milieu	hVSMC	(194)
miR-322/424	−	Smad2/3, MMP-2 and VEGF	Inhibition of inflammation, neoangiogenesis, Promotion of VSMC phenotype transition, and matrix remodeling	hASMC, Mouse Aorta	(148)
miR-712/205	+	TIMP3, RECK	Promotion of ECM remodeling, endothelial permeability, and inflammation	Mouse VSMC, AEC	(195)

h, human; ASMC, aortic smooth muscle cell; ADSC, adipose-derived stem cell; AFB, aortic fibroblast; AEC, aortic epithelial cell; VSMC, vascular smooth muscle cell; TLR4, toll-like receptor 4; CMA1, chymase 1; LCN2, lipocalin-2; CDKN2B, cyclin dependent kinase inhibitor 2B; TXNIP, thioredoxin interacting protein; NLRP3, nucleotide-binding oligomerization domain, leucine-rich repeat and pyrin domain-containing; CMST4, mammalian sterile 20-like kinase 4; PTEN, mutated in multiple advanced cancers 1; FoxO4, forkhead box O4; HI3L1, chitinase-3-like protein 1; GRIA4, glutamate ionotropic receptor AMPA type subunit 4; LYPD3, Ly6/PLAUR domain-containing protein 3; Col1a1, collagen, type 1, alpha 1; Col3a1, collagen, type 3, alpha 1; Col5a1, collagen, type 5, alpha 1; *Eln*, elastin; LRP1, low-density lipoprotein receptor related protein 1; ADMA9, asymmetric dimethylarginine 9; VEPH1, ventricular zone expressed PH domain containing 1; ADAMTS-4, a disintegrin and metalloproteinase with thrombospondin motifs-4; EZH2, enhancer of zeste homolog; TLR2, toll like receptor 2; OLR1, oxidized low density lipoprotein receptor 1; EV, extracellular vesicle; ZIC3, Zic family member 3; MMP-2, matrix metalloproteinase 2; MMP-9, matrix metalloproteinase 9; iNOS, inducible nitric oxide synthase; MCP-1, monocyte chemotactic protein-1; TIMP-3, tissue inhibitor of metalloproteinase 3; KDM3A, lysine (K)-specific demethylase 3A; Smad3, SMAD family member 3; Sirt1, silent information regulator 1; LRP1, low-density lipoprotein receptor related protein 1; VEGF, vascular endothelial growth factor; RECK, reversion inducing cysteine rich protein with lazal motifs.

Another perspective for epigenetic treatment is drawing inspiration from risk factor diseases or other cardiovascular diseases similar to AAA. For example, research has shown that stem cells have potential in treating cardiovascular diseases, with mesenchymal stem cells (MSCs) effectively inhibiting cell senescence in cardiovascular disease (196). Recent results in epigenetic research indicate that upregulating miR-19b-3p can enhance the anti-aging effect of extracellular vesicles from MSCs (MSC-EXO) isolated from AAA patients. This occurs through the regulation of the MST4/ERK/Drp1 pathway, inhibiting mitochondrial fission in VSMCs (177). In addition, there are a number of epigenetic therapeutic drugs in clinical trials in cardiovascular diseases, such as DNMT inhibitors (azacytidine, decitabine, and hydroalazine), HDAC inhibitors (vorinostat, sodium valproate), histone methylation inhibitors (GSK126, EPZ-5676), and histone methylation inhibitors (azacytidine, decitabine, and hydroalazine). Bromodomain and exo-terminal motif (BET) inhibitor (RVX208) (197). They have different effects on cancer and cardiovascular diseases, and may be used as a treatment for AAA in the future.

### 4.3 Future of epigenetic modifications in AAA

Our exploration of epigenetic modifications extends beyond this. In addition to translating established epigenetic modification mechanisms observed in animals into clinical trial directions, other types of epigenetic modifications that have not been well-explored in AAA, such as chromatin remodeling and other types of RNA modifications, are also considered. Two studies on chromatin remodeling focused on the unique subunits BAF60a and BAF60c of the SWI/SNF chromatin remodeling complex, determining that BAF60a promotes epigenetic regulation of VSMC inflammation and BAF60c plays a crucial role in maintaining VSMC homeostasis (198, 199). However, there are still few studies on the specific mechanisms of chromatin remodeling in AAA and even cardiovascular diseases. In the future, more comprehensive studies are expected to make the effects of histone and DNA remodeling at the chromatin level on AAA more clear. With the development of epigenetic technologies, new sequencing and editing methods can reveal complex network regulations of many genes controlled by epigenetic mechanisms. The application of these epigenetic technologies in AAA research can provide a genetic-level understanding of epigenetic modifications as targets and signaling pathways for new drug development and offer critical insights into potential therapeutic strategies for AAA.

However, despite the potential therapeutic advantages of epigenetic therapy, it also comes with limitations. Epigenetic treatments may induce unnecessary changes or off-target effects outside the intended target, potentially leading to adverse reactions or even other diseases. Furthermore, uncertainties remain regarding the long-term impacts and safety of epigenetic therapy, necessitating further research and validation. It is also important to note that most epigenetic research is limited to animal models, *in vitro* studies on human cells, or bioinformatics-based screenings. Effective translation into clinical trials may face obstacles, such as delivery methods or cost and accessibility.

## 5 Conclusion

In the past two decades, there has been rapid progress in the research development of epigenetics. The role of AAA is a complex and multi-faceted issue. A comprehensive understanding of epigenetics in AAA is crucial. On one hand, it involves exploring different targets and pathways, and on the other hand, validating the feasibility of known targets and pathways. Further research is required to confirm the role of epigenetics in the treatment of AAA and the therapeutic potential in AAA animal models.
